# gRNA-SeqRET: a universal tool for targeted and genome-scale gRNA design and sequence extraction for prokaryotes and eukaryotes

**DOI:** 10.3389/fbioe.2023.1217811

**Published:** 2023-08-29

**Authors:** Lisa Simirenko, Jan-Fang Cheng, Ian Blaby

**Affiliations:** ^1^ US Department of Energy Joint Genome Institute, Lawrence Berkeley National Laboratory, Berkeley, CA, United States; ^2^ Environmental Genomics and Systems Biology Division, Lawrence Berkeley National Laboratory, Berkeley, CA, United States

**Keywords:** CRISPR, gRNA, design tool, batch DNA extraction, CRISPR screen

## Abstract

High-throughput genetic screening is frequently employed to rapidly associate gene with phenotype and establish sequence-function relationships. With the advent of CRISPR technology, and the ability to functionally interrogate previously genetically recalcitrant organisms, non-model organisms can be investigated using pooled guide RNA (gRNA) libraries and sequencing-based assays to quantitatively assess fitness of every targeted locus in parallel. To aid the construction of pooled gRNA assemblies, we have developed an *in silico* design workflow for gRNA selection using the gRNA Sequence Region Extraction Tool (gRNA-SeqRET). Built upon the previously developed CCTop, gRNA-SeqRET enables automated, scalable design of gRNA libraries that target user-specified regions or whole genomes of any prokaryote or eukaryote. Additionally, gRNA-SeqRET automates the bulk extraction of any regions of sequence relative to genes or other features, aiding in the design of homology arms for insertion or deletion constructs. We also assess *in silico* the application of a designed gRNA library to other closely related genomes and demonstrate that for very closely related organisms Average Nucleotide Identity (ANI) > 95% a large fraction of the library may be of relevance. The gRNA-SeqRET web application pipeline can be accessed at https://grna.jgi.doe.gov. The source code is comprised of freely available software tools and customized Python scripts, and is available at https://bitbucket.org/berkeleylab/grnadesigner/src/master/ under a modified BSD open-source license (https://bitbucket.org/berkeleylab/grnadesigner).

## Introduction

CRISPR-based genome editing has rapidly become the targeted engineering technology of choice due to its programmability, scalability and near universal application (Jinek et al., 2012; Wiedenheft et al., 2012; Jiang et al., 2013; Ran et al., 2013). The core machinery enabling editing comprises a CRISPR-associated protein (Cas; an endonuclease) and a short guide RNA (a fusion of a variable, target specific sequence and a Cas-specific stem-loop forming sequence required for CRISPR nuclease maturation). Programmability is achieved by complementarity of this variable region to the target DNA, which for Cas binding and double-strand cleavage must be juxtaposed to a Cas-specific sequence termed the protospacer adjacent motif (PAM) (Mojica et al., 2009). This requirement, which for *Streptococcus pyogenes* Cas9, the first to be discovered and the most widely used, is 5′ NGG 3′, constitutes the only limitation in targeting DNA. However, even here alternate Cas endonuclease’s afford flexibility due to different PAMs (albeit with different activities; for example, Cas12a with a PAM of 5′ YTN 3′, induces a 5′ overhang double strand cleavage) (Zetsche et al., 2015; Yamano et al., 2016).

Initial DNA-editing exploited the double-strand cutting induced by Cas9 followed by low efficiency non-homologous end joining (NHEJ) repair mechanisms in eukaryotes yielding loss-of-function mutants. In the absence of NHEJ, a DNA fragment comprising regions of homology on either side of the targeted cut site allows homologous recombination repair to generate a scarless mutation in the genome. Alternatively, CRISPR interference or activation (CRISPRi/a) can be employed to modulate transcription without inducing a break in the genome (Qi et al., 2013; Liu et al., 2019). As the technology has matured additional applications have been developed furthering CRISPR’s editing utility (Pickar-Oliver and Gersbach, 2019; Liu et al., 2021; Nakamura et al., 2021; Wang and Doudna, 2023).

Many web-based and downloadable computational tools are available for gRNA design. These tools typically provide the user with 20 nucleotide sequences flanking a PAM for targeting the specified loci [(Naito et al., 2015; Doench et al., 2016; Concordet and Haeussler, 2018) and reviewed in (Wilson et al., 2018; Alipanahi et al., 2022)]. Automated design tools are particularly useful for the experimental design of constructs involving many, or genome-scale, targets such as needed for CRISPR-screening (Bock et al., 2022), or Perturb-Seq (Dixit et al., 2016). However, many preexisting tools are limited to single or pre-computed model organisms, or, where user-provided genomes can be provided, are specifically optimized for prokaryote genome architecture (i.e., input sequence format does not allow for structural annotations such as multiple chromosomes or specifying intron/exons coding/non-coding regions) (Poudel et al., 2022). CCTop and CHOPCHOP, for example, include the genomes for many organisms (Stemmer et al., 2015; Labun et al., 2019), and additional genomes can be requested by email. Other tools cater to specific communities or groups of organism (Peng and Tarleton, 2015; He et al., 2021).

To overcome these limitations, and to enable universal, organism-agnostic design, we developed the guide RNA Sequence Extraction Tool (gRNA-SeqRET), which is built upon the previously published tool CCTop. CCTop’s standalone version was specifically selected due to its open-source licensing, allowing further development, and the options it provides for design. gRNA-SeqRET allows users to create their own accounts where genomes can be uploaded and securely saved. Designs are scoped by entering a series of criteria into the website, and the data packaged and piped into CCTop. Once the job is complete, the results are accessible for download from the tool’s website. gRNA-SeqRET has two main advantages over other tools: 1) compatible with any user-provided input genome files in GenBank and GFF formats, allowing universal design for both prokaryote and eukaryote genome structures; and 2) functionality to bulk extract specified target DNA regions for scalable repair template design.

## Methods

gRNA-SeqRET employs Flask and jQuery for the web-based user interface (UI) and a PostgreSQL database which maintains track of the user’s genomes and submissions. The tool is composed in Python 3, and utilizes the following open source applications for the indicated tasks: CCTop standalone (Stemmer et al., 2015)—generates the complete list of potential gRNAs for a given pre-processed genome file ranked by predicted cutting score, and a FASTA file with the extracted target region(s); BowTie v1.3.0 (Langmead et al., 2009)—generates the indexes needed by CCTop; The ViennaRNA Package (Lorenz et al., 2011)—generates RNA folding predictions used to evaluate the gRNAs in the CCTop output; BioPython (Cock et al., 2009)—Bio.SeqIO is used to parse genome sequence from GenBank files and convert the sequence to FASTA format; GFFutils v0.11.1 (https://daler.github.io/gffutils/index.html)—provides methods for creating an SQLite database (which contains the processed genome files) from a GFF and searching features annotated in GFF files; GFFtools-GX (https://github.com/vipints/GFFtools-GX)—converts GenBank annotations to GFF3 format; Cromwell—Workflow engine for automating the back-end pipeline (https://cromwell.readthedocs.io/en/stable/).

## Results and Discussion

### gRNA-SeqRET is compatible with any prokaryote or eukaryote genome

**FIGURE 1:**
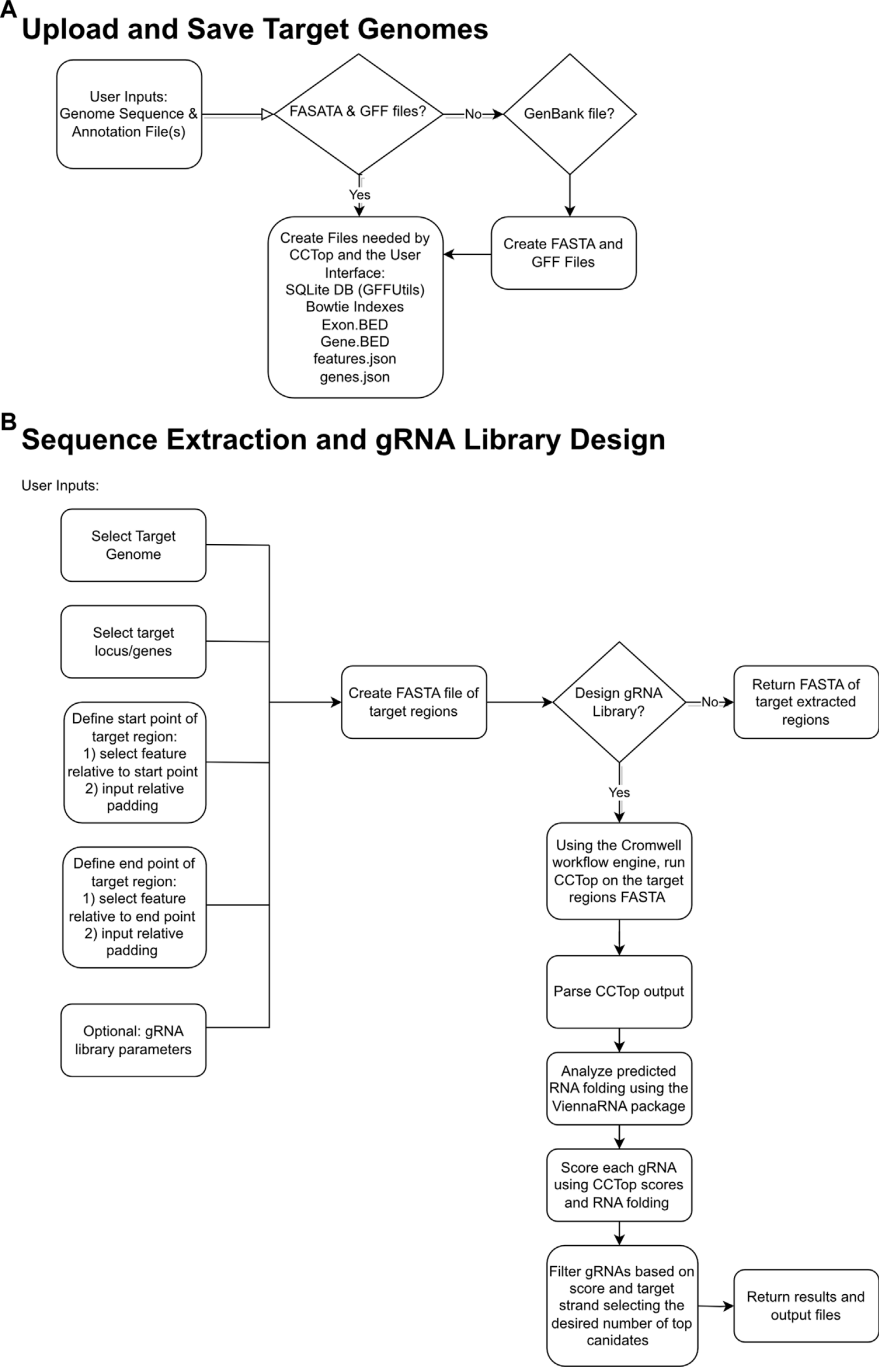
Flowcharts demonstrating relationships between input files, logic choices and outputs for gRNA-SeqRET. Schemas are shown for saving target genomes **(A)** and sequence extraction and gRNA design **(B)**, where diamond boxes represent conditions and rounded rectangles represent tasks and files.

The goal of gRNA-SeqRET is to provide an intuitive web-based user interface for the design of gRNA sequences and custom sequence extraction from user-provided genomes. Figure 1 provides overview schematics of the general tool workflow, focusing on both the processing of the uploaded genome data and the subsequent extraction and guide RNA designs.

The gRNA-SeqRET application accepts either FASTA or GenBank file types for a given organism’s genome. New users of gRNA-SeqRET are required to register an account, which serves two purposes. Firstly, this allows uploaded genomes to be securely maintained on the server (genome files need only be uploaded once, and no files are accessible to other investigators). Secondly, this approach enables asynchronous use of the tool, by which an uploaded genome can be saved and processing (which takes many minutes) can be performed in the background. Files can be uploaded in compressed format (.zip or .gz), though must only contain a single file. If a FASTA file is uploaded the user will be prompted to additionally provide annotations in the format of a GFF3 file (Figure 1A; Table 1). The GFF format is preferred due to the complexities of converting GenBank to GFF, especially for eukaryotes. The uploaded files are processed in order to generate the required input files for CCTop: Bowtie indices, Exon and Gene BED and json files. Specifically, indices are generated using bowtie-build, and the BED files are created using a python script provided with CCTop’s standalone source code. Additionally, a searchable SQLite database is created using GFFUtils, which contains the annotations extracted from the uploaded GenBank or GFF file. This is used to generate JSON files that list the annotated genes or locus IDs and the features (e.g., CDS, exons, *etc.*) enabling the population of genome feature menus (see below).

**TABLE 1:** Input files, parameters and results for gRNASeq-RET.

Item (URL)	Field	Description, page location, default parameters and input options
Input file(s) (https://grna.jgi.doe.gov/save_genome.html)	Genome data	Requisite genome data providing sequence data and feature coordinates. Can be provided as either a GenBank or both a FASTA and GFF (GFF is preferred, especially for eukaryotes)
Target region selection (https://grna.jgi.doe.gov/create_step1.html)	Design name	Unique user provided name that will become the job ID
	Target genome	Uploaded, processed genomes can be selected from the dropdown menu
	Target regions	Individual, or groups of loci in the menu detected from the input annotations can be copied to select, or left blank for genome-scale targeting
	Limit regions	Target regions can be limited to coding, non-coding or both
	Start and end point definitions	Defines the number of base pairs upstream and downstream of a feature in the genome file
	Guide RNA design	Opens options for gRNA design for the specified target regions
	PAM	Specifies the Cas-specific protospacer adjacent motif (PAM)
	Scaffold sequence	Defines the scaffold sequence; default is the canonical hybrid scaffold and contributes to the folding score generated by gRNA-SeqRET
	Target site length	Specifies the spacer region length; default is 20
	Max mismatches offsite targets	Specifies the maximum number of mismatches that will be considered an off-site target and will be excluded
	No. gRNAs	Specifies the number of guide sequences returned to the output file per target region. Note, unlike CCTop, which returns all possible guides, gRNASeqRET will only output this specified number, ranked by CCTop’s predicted cutting score (default is 3)
	Target strand	Optionally filter results based on whether the gRNAs are on the coding or non-coding strand
Job results (https://grna.jgi.doe.gov/design_list)		Lists all complete and running jobs

**FIGURE 2:**
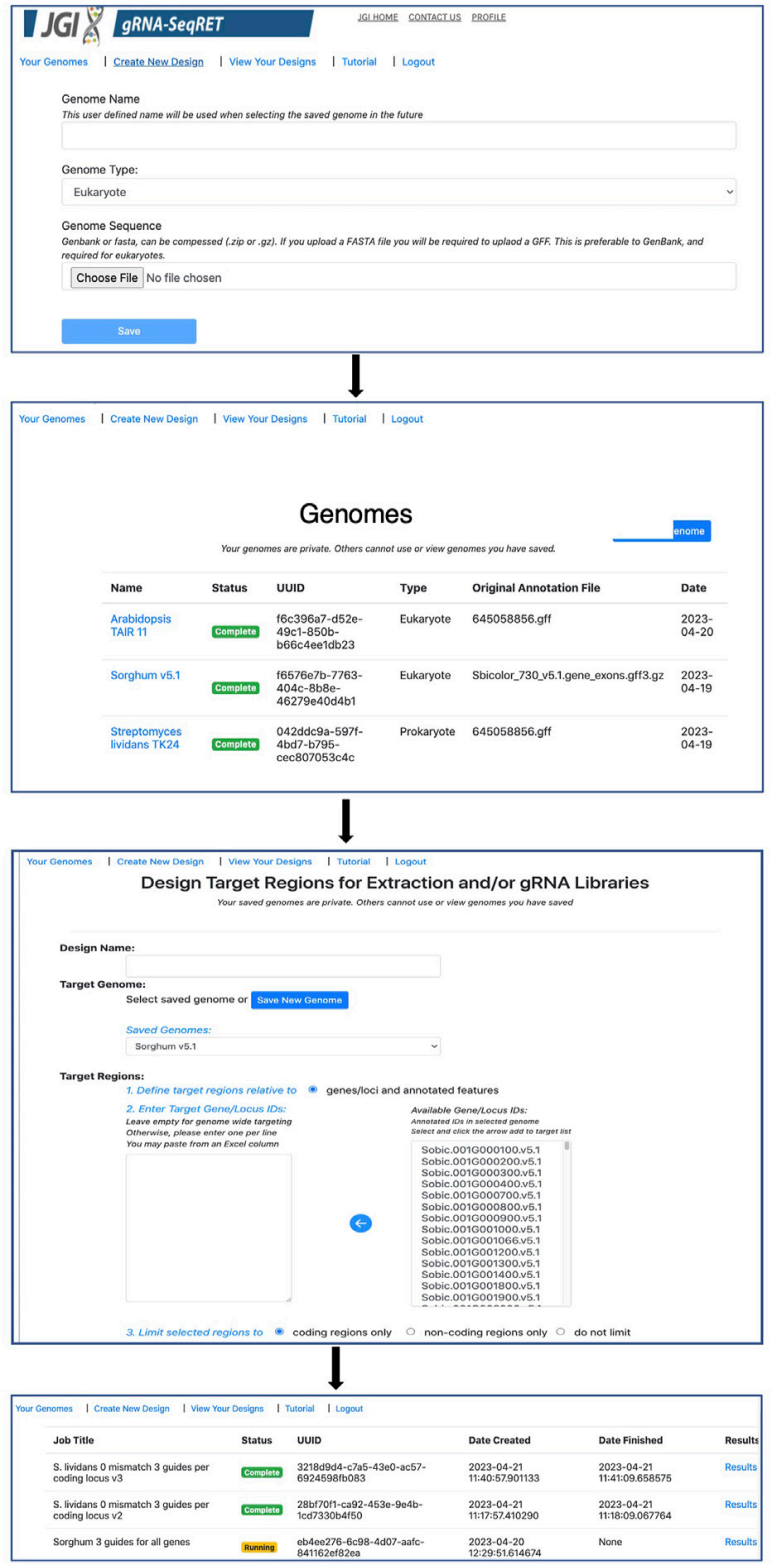
Walkthrough of the gRNA-SeqRET design process.

Processing newly uploaded genomes typically takes around a minute for a small prokaryotic genome, and approximately 5–10 min for a large complex eukaryote (e.g., a plant) genome. Once generated, the processed files are preserved in a user’s individual account and are inaccessible to other users. Consequently, this step needs to be performed only once per genome, and once complete, is ready for initiating designs. All processed genomes will be available in the “Your Genomes” page of gRNA-SeqRET with the status confirmed as complete (Figure 2).

### gRNA-SeqRET automates feature batch extraction and guide RNA design

Once preprocessing is complete, designs and sequence extractions can be performed on genomes uploaded by selecting the Create New Design tool in the top left corner of the menu (Figures 1B, 2). After entering a name for the design, which will become the job title, and selecting a genome from the drop-down menu, the user will specify the loci to be targeted. All locus IDs identified in the provided genome input files will be automatically populated in the menu on the right of the page; from here, individual loci can be selected or pasted, or if left blank all loci will be applied for genome-scale targeting.

Next, the precise regions to target must be provided, enabling batch programming for 1) extraction of genomic sequence data within defined regions and 2) design of gRNA sequences within these defined areas (described in Table 1). Specific use-case examples for various options are described below. Target region definitions are enabled at the nucleotide-level resolution relative to defined features in the input files by entering details into subsequent menus (Figures 1B, 2). This is done by firstly stipulating whether coding, non-coding or both coding and non-coding regions should be included, and secondly by entering the number of bases upstream or downstream of feature reference points to target. The nature of features (defined as reference points in gRNA-SeqRET menus) available in these menus is dependent on those provided in the genome input file; for prokaryotes this will generally be limited to coding sequences (CDSs) and non-coding sequences, but eukaryote GFF files may provide broader options, such as introns and exon coordinates, depending on the extent of structural annotations in the uploaded annotations file. The tool warns if incompatible inputs are provided (for example, an error message will appear and the submit button will be disabled if in the first section the default “coding regions only” is selected as well as a region upstream of the loci coding region). To provide maximal flexibility of region selection, a user can define the number of bases up- or downstream of two independent reference points, constituting the start and end points of the target regions within the genome. Bases upstream of a feature are designated as being negative, and positive numbers indicate the number of bases downstream of this feature (e.g., a start codon). A dynamically generated schematic illustrating the selected area aids the user by updating in real time as entries are made (Figure 2).

This sequence selection feature has multiple utilities, as it enables genome-scale, batch extraction of defined regions. One example would be for homology arm design, as required for CRISPR repair template design, which can then be bulk-downloaded as a FASTA file. Additionally, defining sequence regions also serves to specify the regions with which to target gRNA design, such as targeting specifically upstream (but not so far upstream as to enter the next 5’ coding genome feature) of a gene for promoter-region CRISPRa guides, as described below. Furthermore, batch extraction of defined regions of sequence has functions beyond CRISPR, for example, defining homology arms for homologous recombination (HR)-mediated gene disruptions, or for bulk extraction of sequence upstream of coding regions for promoter libraries. At this stage the defined sequences can be downloaded as a FASTA file, and/or the tool can proceed to gRNA design.

If the gRNA design option is selected, additional fields are made available (Figure 2). Several of these parameters, including the specific PAM, scaffold sequence, target site length and number of allowed sequence mismatches are necessary inputs for CCTop, and detailed information is provided in the publication describing that tool (Stemmer et al., 2015) and summarized in Table 1. Beyond CCTop prerequisites, gRNA-SeqRET additionally asks for the desired number of gRNAs per target and a preference for targeted strand. These last two fields provide the criteria serving to limit the complete CCTop output to just those matching the user’s requirements; i.e., the number of guides specified in the “Number of guide RNAs per gene/custom feature” input box. For example, entering “3” in this field will yield the top 3 gRNAs as determined by CCTop’s predicted cutting score and RNA folding predictions for the protospacer and scaffold. Clicking “submit” executes the job, which can range from seconds for low numbers (e.g., <10) of target loci to several minutes for genome-scale (i.e., all loci) in prokaryotic genomes, to several hours for genome-scale jobs in very large eukaryotic genomes (a submission comprising design of 3 guide RNAs for every coding region of the Araport11 annotation of the *Arabidopsis thaliana* genome (Cheng et al., 2017) ran ∼12 h). The “View Your Designs” page lists all completed and running jobs and provides a results link to the job output (Figure 2). Clicking this link provides a summary of the job parameters as well as the output files for downloading. “Results” provides a comma separated values (CSV) file containing all guide sequences, providing a unique name (the locus name appended by the CCTop designation), the start and end chromosomal coordinates, strand orientation, sequence and specific PAM. “Target regions” provides a FASTA output of all targeted sequences, and the Report contains a list of loci where the desired number of gRNAs was not found, which can be used to run another design round with an altered sequence targeting criteria if desired. Clicking on “All output files (.tar.gz)” will download an archive of all files described above, as well as a file called “scoring.log” which contains all statistics and predicted cutting scores associated with each gRNA. Output files are maintained within the user’s individual account for 3 months. Additionally, users can make their designs public by clicking the “Make Publicly Available” option. Clicking this will open a new page where fields describing the purpose of the library and the target organism NCBI taxonomic ID can be completed, and the designs will be accessible to all users in the “Public Designs” link.

### Applicability of a given gRNA library to other closely related genomes

**FIGURE 3:**
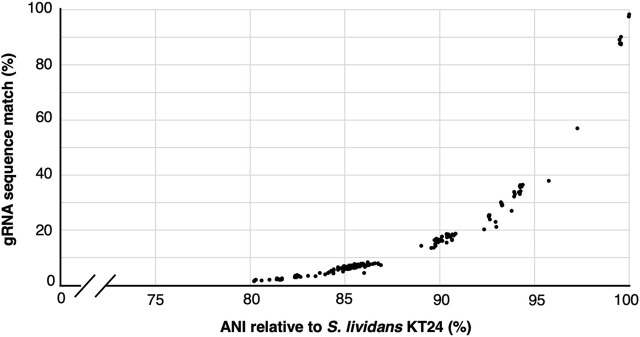
gRNA sequence applicability reduces rapidly with genome distance. Correlation between the percentage of 22,741 gRNAs designed to target *S. lividans* TK24 that align perfectly to 192 related organisms showing an average nucleotide identify (ANI) of ≥80%.

Cost reductions and availability of high-variant oligonucleotide pools and high throughput sequencing has enabled the construction of gRNA libraries to become powerful and increasingly accessible approaches for rapid gene function interrogation (Schwartz et al., 2019; Bock et al., 2022; Cooper et al., 2022; Shi et al., 2022; Trivedi et al., 2023). Nevertheless, the library assembly and sequencing-based quality control (ensuring representation of all variants and possible skews in the population) can represent a significant endeavor and investment. Since a single constructed cloned library yields sufficient material for many tens of independent experiments, we wondered how applicable a given gRNA library would be to other closely related species. To address this, we first used gRNA-SeqRET to design a genome-scale gRNA library targeting all coding regions in the Actinomycetes *Streptococcus lividans* TK24. gRNA-SeqRET was employed to report 3 gRNAs per target region, resulting in 22,641 total spacer designs (Supplementary Table S1). We turned to the Integrated Microbial Genomes and Microbiomes (IMG) (Chen et al., 2023) database and queried for all genomes characterized with an average nucleotide identity (ANI) ≥80% relative to *S. lividans* TK24, yielding 192 genomes (Supplementary Table S2). While an ANI is a statistic generated from the complete genome sequence, and we specifically searched for gRNA sequences in coding regions only, this approach enabled us to retrieve highly similar genome sequences. We then searched the genomes of each of these 192 microbes for perfect matches to each of the ∼22 thousand 20mer sequences (plus PAM; Supplementary Table S3), providing a sense as to how applicable a given gRNA library could be to alternate closely related organisms. Unsurprisingly, guide sequence alignment reduces dramatically with genome distance (Figure 3). While the fraction of guide sequences with perfect alignment remains high (∼90%) for genomes with very high similarity (i.e., ANI >98%), this drops to ∼45% in genomes with and ANI of 95% (Figure 3; Supplementary Table S3). Nevertheless, since organisms belonging to the same species typically exhibit an ANI of 95%, this analysis demonstrates a pooled gRNA library designed to one organism could be of value to strains within a species, and perhaps to other closely related species (Konstantinidis and Tiedje, 2005), although this will depend on the specific species. Worth noting though is with increased taxonomic distance and genetic drift, off site targeting may also increase and confound the data. Additionally, we found that many of the 192 genomes contained several hundred occurrences of multiple matches per guide sequence, indicating a perfect sequence alignment in an off-target location (Supplementary Table S3).

## Future directions

During our internal use of the present version of gRNA-SeqRET, we have identified several areas we are considering for future enhancement. The first of these builds upon the possible re-use of guide RNAs in multiple organisms, by adding an option to provide multiple genomes so that the application can filter out possible gRNAs that occur in both genomes but without off-target events. Another is to enable a user to provide custom annotations allowing regions to be targeted or extracted in addition to those defined in the genome input file. Finally, we may develop an option to target specific alleles in polyploid genomes.

## Data Availability

The original contributions presented in the study are included in the article/Supplementary Material, further inquiries can be directed to the corresponding author.
